# Everybody's Page

**Published:** 1892-06-18

**Authors:** 


### Everybody's Page.

The Editor of *The Gentlewoman* put to his readers the question—"Do Women Desire to Vote for Members of Parliament?" and asked them to vote thereon. The ballot, which has been open for several weeks, closed on Monday with the following result: 8,301 voting "Yes," and 1,158 voting "No," in answer to the above question. This is an interesting and useful indication of what Mr. Gladstone called the "womanly mind" of the kingdom upon the subject.

Chicago is so eminently characteristic in many ways of Americans that it has been well chosen as the site for the World's Fair. As a correspondent to the *Times* pointed out recently, that City deals with "bigness" of every kind, boasting of the largest shop in the world, the largest elevators, and the largest meat-packing establishment. As size is everything in the eyes of the American, they are very proud of the phœnix which arose from her ashes of 1871 with such rapidity, and the establishment there of the largest exhibition in the world should bring the inhabitants to the summit of their ambition.

Some of our readers are probably not aware that meningitis can be cured by the application of glycerine to the brain through the nostrils. Of the efficacy of this simple remedy a theologian, electrician, and engineer is perfectly satisfied. A wooden hatch fell on his head, and a brass handle attached cut the scalp into the bone. Subsequently symptoms of inflammation of the brain showed themselves, and glycerine was injected through the nostrils and the alarming symptoms disappeared. No doubt the happy combination of faith, and electrical and engineering skill had something to do with the action of the glycerine. Why should not glycerine soothe a ruffled temper just as oil smooths troubled waters!

It was foretold of machinery when first introduced to our manufacturing centres, that it would oust all manual labour. But like most prophecies the forecast has been proved false. Now it is electricity which is to be the great labour saving appliance. Enthusiastic believers in it look forward to an enormous expansion of its uses in the domestic circle. If we can cook by electricity why should not our carpets be swept, and our rooms cleaned by it. Nature has already presented us with electric eels, why should not we have electric servants? Here is a vision of untold joy and delicious economy to the harassed housekeeper whose weekly books are an agonising torture to her.

The attempt to cure drunkenness by statute does not seem to have met with much success in the various States of America. But the legislature is still undaunted, and in Iowa it is now proposed to establish "County Boards of Dipsomania," with power to supervise the treatment and cure of inebriates. Unfortunately it is one thing to supervise the cure of drunkenness, another to accomplish that desirable end.

In the opinion of the *Medical Record* the ancient controversy over the identity of membraneous croup and diphtheria seems likely to take a new phase, and one which may revive the dual theory of the disease, which has of recent years been practically abandoned. Our readers will not be surprised to hear that the origin of this probable change of front is bacteriology. Yet the only conclusion to be drawn from observations lately made is that while some cases of membraneous croup are true diphtheria, others are not. This, after all, leaves matters very much where they were.

An original scheme has been propounded by a correspondent in *Truth*, which, if carried out, will bring the millennium within the reach of all. Like many brilliant ideas, the scheme is an adaptation. The penny-in-the-slot principle is to rid us of our intricate poor law system, and the machine which now enriches us with toffee and wax vestas is to solve the problem of labour tests for casuals. The "Knight of the Road" is not asked to do such an unreasonable act as to put a penny in the slot; if he will only turn a handle a penny will come out of the slot. The power thus expended, as the correspondent points out, can be utilised in innumerable, and far more profitable ways than oakum-picking. Here is a glorious future for the unemployed, whose ranks we mean to join the moment the scheme is matured.

The Society for the Protection of Children in France at last appears to have been able to arouse something more than apathetic interest in the question of the stationary nature of the population of that country. Statesmen are beginning to give the matter their serious attention, with the result that the State is intervening in the management of infants. It may almost be said that the State is undertaking the work of a baby-farm, for nurses are now forbidden by law, at any time or under any pretext whatever, to use nursing-bottles provided with a rubber tube in the rearing of infants confided to their care. The proper or legal length of an infants' garment has not yet been settled, we believe, but no doubt it will shortly engage that attention at the hands of the French ministry, which a question of such national importance deserves.

